# Glycation changes molecular organization and charge distribution in type I collagen fibrils

**DOI:** 10.1038/s41598-020-60250-9

**Published:** 2020-02-25

**Authors:** Sneha Bansode, Uliana Bashtanova, Rui Li, Jonathan Clark, Karin H. Müller, Anna Puszkarska, Ieva Goldberga, Holly H. Chetwood, David G. Reid, Lucy J. Colwell, Jeremy N. Skepper, Catherine M. Shanahan, Georg Schitter, Patrick Mesquida, Melinda J. Duer

**Affiliations:** 1 Department of Chemistry, University of Cambridge, Lensfield Road, Cambridge, CB2 1EW UK; 2 Babraham Institute, Cambridge, CB22 3AT UK; 3 Cambridge Advanced Imaging Centre, Department of Physiology, Development and Neuroscience, University of Cambridge, Downing Street, Cambridge, CB2 3DY UK; 4 BHF Centre of Research Excellence, Cardiovascular Division, James Black Centre King’s College London, 125 Coldharbour Lane, London, SE5 9NU UK; 5 Automation and Control Institute (ACIN), TU Wien, Gusshausstrasse 27-29, A-1040 Vienna, Austria; 6 Department of Physics, King’s College London, Strand, London, WC2R 2LS UK

**Keywords:** Nanoscale biophysics, Biophysical chemistry, Chemical modification, Atomic force microscopy, Electron microscopy, Chemical biology, Biophysics, Structural biology

## Abstract

Collagen fibrils are central to the molecular organization of the extracellular matrix (ECM) and to defining the cellular microenvironment. Glycation of collagen fibrils is known to impact on cell adhesion and migration in the context of cancer and in model studies, glycation of collagen molecules has been shown to affect the binding of other ECM components to collagen. Here we use TEM to show that ribose-5-phosphate (R5P) glycation of collagen fibrils – potentially important in the microenvironment of actively dividing cells, such as cancer cells – disrupts the longitudinal ordering of the molecules in collagen fibrils and, using KFM and FLiM, that R5P-glycated collagen fibrils have a more negative surface charge than unglycated fibrils. Altered molecular arrangement can be expected to impact on the accessibility of cell adhesion sites and altered fibril surface charge on the integrity of the extracellular matrix structure surrounding glycated collagen fibrils. Both effects are highly relevant for cell adhesion and migration within the tumour microenvironment.

## Introduction

Collagen fibrils are central to the molecular organization of the extracellular matrix (ECM) and thus to defining the cellular microenvironment^1,2^. Cells bind to specific GXOGER sequences on collagen fibrils via non-covalent interactions with transmembrane integrin receptors for both adhesion and migration purposes^3–10^, signal transduction via integrin-collagen binding being relevant for cell growth and differentiation^11^. The extent of integrin binding to collagen – and thus the strength of signalling – is dependent on the degree of exposure of the relevant GXOGER molecular sequences on the collagen fibril surface, which in turn depends on the arrangement of collagen molecules within the fibrils. Non-covalent interactions between specific amino acid sequences in collagen molecules and other extracellular matrix (ECM) proteins also direct the organisation of molecules around collagen fibrils and so are crucial in both the initial self-assembly of the ECM and in maintaining its structural integrity. Thus, the physicochemical and structural state of collagen fibrils is highly relevant to cell growth and mobility and to the overall spatial organization of the extracellular matrix networks.

Collagen fibrils are subject to non-enzymatic glycation reactions because they are relatively accessible to exogenous sugars and other aldehydes. Glycation is a spontaneous non-enzymatic reaction between carbonyl groups of reducing sugars and amine groups of proteins, lipids or nucleic acids, occurring in all living systems^12,13^. The glycation reactions between collagen and sugars are highly complex, with many possible sugar-adduct intermediate products and further reactions steps to form so-called advanced glycation endproducts (AGEs), the latter of which includes intermolecular crosslinks^14,15^. The glycation reaction begins with Schiff base formation at lysine sidechain amines and then Amadori rearrangement to a variety of sugar adducts of Lys through a range of facile intramolecular rearrangements^15–20^. These initial products are susceptible to oxidation and fragmentation to smaller sugar aldehydes such as methylglyoxal which are highly reactive and generate further products which can react with both Lys and Arg sidechains^21^. Thus the initial glycation reaction sets off a cascade of chemical reactions leading to a distribution of a multitude of products^13,22–25^.

Until very recently, the focus of collagen glycation research has been on AGE crosslinks. Glycation of extracellular matrix collagens in *in vivo* tissues^21,24,26–28^ is well known to detrimentally stiffen collagen fibrils^21,29,30^. Correlations between increases in the number of advanced glycation endproduct (AGE) crosslinks per collagen molecule and increasing collagen fibril stiffness led to the belief that AGE crosslinks cause the stiffening^13,25,29^ and further, that AGE crosslinks are the predominant source of both the mechanical and biological consequences of collagen glycation. However, these studies quantified only AGE crosslinks and did not characterise or quantify the other glycation products that co-exist with AGEs. It has recently been shown that the majority of the glycation products for glucose glycation of collagen *in vivo* are monovalent sugar adducts of lysine^31^ and that in fact the total number of crosslinks per collagen molecule is decreased in glucose-glycated collagen fibrils, because glycation leads to loss of enzymatic crosslinks^31^. These recent new insights imply that monovalent glycation products play a very significant role in fibril stiffening, rather than AGE crosslinks. Thus, the key question of how and why glycation causes detrimental changes in collagen fibril physical properties has to be re-opened.

The nature of the glycating sugars in the ECM is an equally important question. In ageing and diabetes, glucose is the main glycating sugar and for this reason, glucose has been the focus for understanding the consequences of collagen glycation. However, there are numerous other sugars present in blood plasma that can potentially also play a role. Ribose and ribose-5-phosphate (R5P) whilst present in lower concentrations than glucose in blood plasma (0–17 μM for ribose^32^ and 13 μM for R5P^33^ in blood plasma), both react considerably more rapidly with protein amine groups than glucose, ~150 times faster in the case of R5P^17,34^, meaning that their glycation products can be highly relevant in collagen glycation. The biosynthetic origin of R5P is the pentose phosphate pathway (PPP). PPP activation is the hallmark of rapidly proliferating cells including pluripotent stem cells, cells in the blastocyst^35^ and cancer cells^36^; R5P concentration has been shown to increase 2–10 fold in some breast tumour cell lines, for instance^37^. Where there is additionally cell necrosis, as in the tumour setting, there is the possibility of even higher local R5P concentrations in the extracellular fluid. We have recently shown that the majority of the collagen glycation products for ribose and ribose-5-phosphate (R5P)^16,38^ are, as for glucose glycation, monovalent sidechain modifications^16,38^, primarily sugar adducts and that the dominant AGEs are carboxymethyllysine (CML), carboxyethyllysine (CEL) and norpronyllysine (see Fig. S1) consistent with previous work on R5P glycation of amino acids^17^. The propensity of R5P to glycate collagen^17^ may thus significantly modify the cell microenvironment where is PPP is highly activated, such as in cancer.

Importantly, glycation of collagen fibrils has been shown to have significant biological consequences. Cells adhere less well to glycated collagen fibrils, proteoglycan binding to glycated collagen is diminished^39^ and glycated collagen activates the receptor for AGE products (RAGE) in cells, which plays roles in vascular disease^12,40^, diabetic complications^41^ and cancer^42^. Whilst physical stiffness of collagen fibrils may be responsible for some part of these effects, there is as yet insufficient evidence to conclude this. Equally possible is that collagen glycation affects cell adhesion by altering the accessibility or structure of collagen fibril integrin binding sites. However, the effects of glycation on the molecular arrangements within collagen fibrils has been relatively little studied^43^.

**Figure 1:**
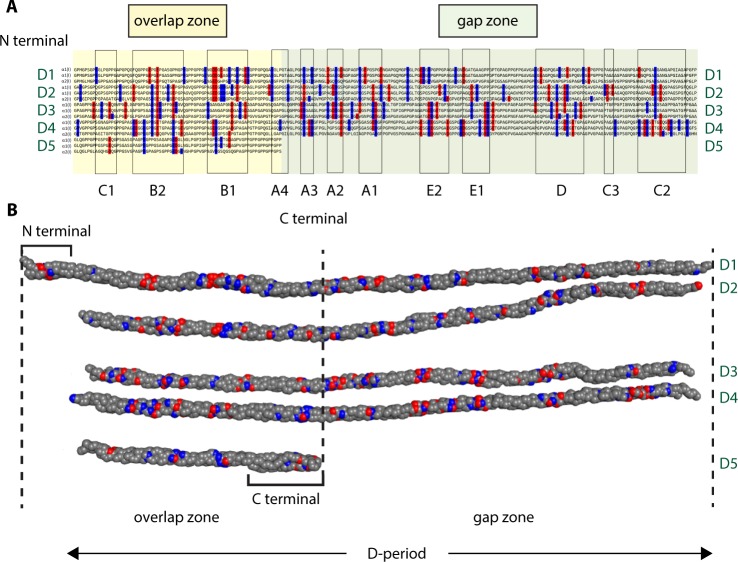
(**A**) The consensus sequence for mammalian collagen type I arranged by D period, the expected arrangement in *in vivo* collagen fibrils^44^ showing the distribution of charged residues along the sequence. The collagen molecule consists of three peptide chains, two α1 and one α2 chain, each staggered by one residue with respect to each other and twisted into a triple helix for the majority of their length (stagger not shown because it is unknown whether the heterochain, a2, is in the leading, middle or trailing position within the triple helix). The schematic corresponds to the repeat unit cell in a collagen fibril. This arrangement leads to the so-called overlap zone, where all five D periods overlap and the gap zone where the short D5 period leads to a gap or hole in the fibrillar structure. Note that only the triple helical region of the molecule is shown and that the non-helical N- and C-terminal telopeptide sequences are not shown. The charged amino acids are highlighted in red (negatively charged residues) and blue (positively charged residues). The regions enclosed in rectangular boxes indicate collagen sub-bands as determined by TEM^44^. These regions contain an abundance of charged residues, both positively and negatively charged (note that the schematic does not represent the exact alignment of charges because the chain stagger within the triple helix is not included here and because the triple helix does not have constant pitch over its length). (**B**) 2D representation of the collagen type I fibril structure determined from x-ray diffraction analysis (3HR2) for one D period with N- and C-terminal telopeptides included^57^, showing the spatial distribution of charged residues. Charged residues highlighted as in (**A**). Note that only Cα atoms are shown and that the spheres representing them are not indicating van der Waals radii.

Molecular organisation within collagen fibrils (Fig. 1) is determined by numerous non-covalent interactions between collagen molecules, in particular charge-charge interactions^44–47^. Interestingly, Lys positions in fibrillar collagens are the most conserved of all residues, even more so than proline/ hydroxyproline, the major constituents after Gly. Glycation of Lys sidechains alters both the length of the sidechain by addition of sugar adducts and the charge distribution on the sidechain^38^, both parameters expected to disrupt molecular organisation within collagen fibrils. Altered molecular packing in collagen fibrils can be expected to affect the accessibility of integrin binding sites and the molecular dynamics of those binding sites, both of which are relevant to collagen-mediated integrin signalling. Alterations in both molecular and fibrillar charge distributions are also expected to affect collagen fibril interaction with surrounding ECM components, thus having a knock-on effect on the ECM network as a whole, significantly altering the cellular microenvironment. There is a need to understand the consequences of R5P glycation of collagen on the physicochemical properties of collagen fibrils and on the molecular arrangements within them in order to generate new hypotheses about how R5P collagen glycation may impact cell adhesion and migration and the integrity of the wider extracellular matrix structure, particularly in the context of cancer. Here, we explore the effects of R5P glycation on collagen fibril molecular organization and fibril surface charge to gain understanding of cell microenvironment in the presence of this avid glycator.

## Results

R5P-glycated collagen fibrils were generated as previously described^38^ by incubating bovine Achilles’ tendon collagen type I and either U-^13^C-R5P (for NMR verification of R5P glycation products) or unlabelled R5P (for all other experiments) for six weeks to ensure equilibrium for all reactions is reached. The distribution of R5P glycation products was verified to be similar to that previously described^38^ by 1D ^13^C and 2D ^13^C-^13^C correlation NMR spectroscopy (Fig. S1), namely the dominant products are monovalent sugar adducts. ^31^P NMR (Fig. S1) showed that there was no significant phosphate in R5P glycated samples, and thus that the glycation reactions result largely in the removal of the R5P phosphate group as expected^17^. The dominant AGE observed by NMR spectroscopy was carboxymethyl lysine, consistent with previous observations^17^ and LC-MS confirmed the presence of pentosidine as the dominant AGE crosslink (Fig. S1). Native collagen is subject to glucose glycation and thus we also measured the extent of pre-existing glucose glycation in the bovine tendon collagen by LC-MS and any changes after R5P glycation (see Table 1 below). Pre-existing glucose glycation of Lys is present on 0.47 ± 0.02 Lys per collagen molecule and on 0.15 ± 0.01 Hyl per collagen molecule for the bovine tendon collagen samples used here. This drops to 0.17 ± 0.05 Lys per collagen molecule after R5P glycation treatment, whilst the Hyl glucose glycation is largely unchanged (0.11 ± 0.02 Hyl per collagen molecule). These results suggest that R5P glycation may displace existing Lys glucose glycation, but that Hyl glucose glycation is largely unaffected by R5P.

**Table 1:** LC-MS analysis of enzymatic cross links and pre-existing glucose glycation for type I collagen used in this work and ribose-5-phosphate glycated collagen per mole of collagen (mole/ mole(Pro + Hyp)/669); standard deviations in brackets.

sample	HLNL	DHLNL	LNL	PYD	DPD	Pre-existing glucose-glycated Lys	Pre-existing glucose-glycated Hyl
Unglycated	1.06 (0.07)	0.140 (0.002)	0.025 (0.002)	0.65 (0.04)	—	0.47 (0.02)	0.15 (0.01)
R5P (50 mM) glycated	0.4 (0.1)	0.09 (0.07)	0.07 (0.04)	0.38 (0.15)	0.11 (0.04)	0.17 (0.05)	0.11 (0.02)

DPD levels in unglycated collagen were too low for measurement. (HLNL = hydroxylysinonorleucine; DHLNL = dihydroxylysinonorleucine; LNL = lysinonorleucine; PYD = pyridinoline; DPD = deoxypyridinoline).

### Glycation changes molecular ordering in collagen fibrils

**Figure 2:**
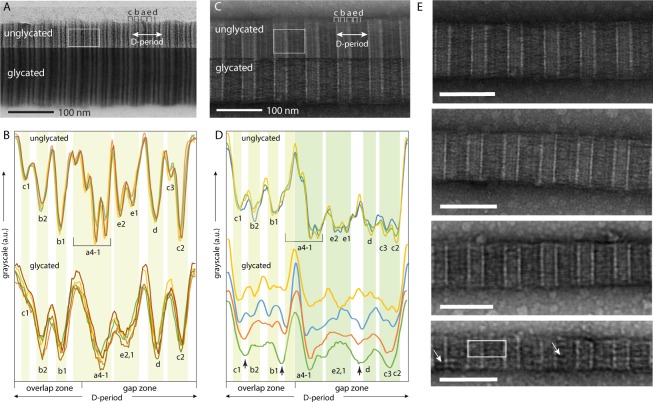
**(A,B):** Bright-field (BF) TEM of positively-stained (uranyl acetate) collagen fibrils. **(A)** Comparison of TEM images of glycated (top) and unglycated (bottom) collagen fibrils. The differences in overall image brightness between glycated and unglycated are arbitrary and used here to distinguish the images of the glycated/ unglycated fibrils. The sub-bands for one fibril D-period are labelled^44^ for the same fibril D-period definition as in Fig. 1. The white rectangle indicates the typical region size over which the densitometry plots in B are calculated. **(B)** Densitometry plots (image intensity profiles) for representative D-periods for glycated and unglycated collagen fibril images, examples of which are shown in (**A**). The expected positions of the sub-bands for unglycated fibrils are indicated. **(C,D):** Bright-field (BF) TEM images of negatively-stained (uranyl acetate) collagen fibrils, equivalent to those in (**A,B**). In (**D**), the densitometry plots for unglycated collagen are from randomly selected D-periods on a single collagen fibril; plots are highly similar for all unreacted fibrils examined. Those for the glycated collagen are from the glycated collagen fibrils in **(E)** for a single D-period (as exemplified by the white rectangle). The uppermost densitometry plots for glycated collagen fibrils in (**D**) (blue and green) are from thicker fibrils (top two fibril images in (**E**) and the bottom two densitometry plots (yellow and brown) are from thinner fibrils (bottom two fibril images in (**E**). Dotted lines in densitometry plots in B and D indicate where relative image intensity is significantly reduced for glycated fibrils compared to unglycated fibrils. Scale bars in (**E**) are 100 nm.

We hypothesised that glycation alters the molecular arrangement within collagen fibrils through alteration of Lys and possibly Arg sidechain size and charge distribution. We examined the effects of collagen R5P glycation on the molecular ordering in collagen fibrils using TEM imaging. Positive staining with uranyl acetate of control collagen fibrils that had not been reacted with R5P gives characteristic patterns of thin, stained sub-bands in TEM images (termed “sub-bands” to distinguish them from the gap/ overlap zone differential staining observed in negative staining of collagen fibrils) due to the alignment of charged sidechains in the fibril molecular organisation (Fig. 2A)^44^. Densitometry plots across fibril D-periods in these TEM images (Fig. 2B) show that glycation with R5P leads to a loss of definition of the a and c sub-bands (gap/ overlap interface) and the gap zone e sub-bands visible as a broadening of the peaks associated with the respective sub-bands. All staining at the a4 sub-band position is lost in R5P-glycated fibrils and that at the c3 position substantially reduced. Within the overlap zone, broadening of the c1 and b1 sub-bands is also apparent, though to a slightly lesser degree than the broadening of the affected gap zone sub-bands. The loss of definition of sub-bands with R5P glycation indicates that the bands of molecular charge become disordered or less sharply defined as a result of glycation.

In images of negatively-stained collagen fibrils, gap zones appear darker (more stained) and overlap zones lighter (less stained)^44^, because the gap zone is atomically less dense compared to the overlap zone and so takes up more stain. The gap/ overlap zone differential staining pattern allows precise measurement of the D-period length for individual fibrils. For unreacted collagen fibrils, the mean D-period length was measured to be 67.1 nm with standard deviation (SD) of 0.7 nm (N = 15) and for R5P-glycated fibrils, 66.6 nm with SD of 1.5 nm (N = 12). Thus, although the average D-period length is not significantly altered by glycation, there is a much greater variability of D-period lengths in glycated fibrils.

The sub-banding patterns in images of negatively-stained fibrils (Fig. 2C) partly arise from the alignment of charged sidechains (Fig. 1) and partly from the locations of bulky versus less bulky residues^44^. The density profiles extracted from images of negatively-stained R5P-glycated collagen fibrils (Fig. 2C,D) exhibited broadening of most sub-bands relative to those from images of control fibrils that had not been reacted with R5P. Additionally, loss of intensity of the gap zone d sub-band for glycated fibrils (Fig. 2D) was consistently observed. There were small differences in the effect of R5P glycation between thick (200–300 nm diameter) and thin (<150 nm diameter) collagen fibrils. For thick fibrils, the c1 and b2 sub-bands are significantly reduced in intensity compared to non-reacted fibrils, and new sub-band intensity appears between the c1 and b2 sub-band positions, whilst for thin fibrils, the c1 and b2 sub-bands are relatively unaffected in intensity or width, in contrast to the rest of the sub-banding.

In summary, the observed alterations in the sub-band intensity pattern in R5P-glycated collagen compared to unglycated fibrils seen after positive and negative staining for TEM imply both charge and molecular structural rearrangements particularly in the fibril gap zone and gap/ overlap interface arising as a result of R5P glycation.

### R5P glycation lowers the collagen fibril surface charge

We next hypothesised that R5P glycation of collagen fibrils modifies the fibril surface charge^43^. We used two independent methods of assessing change in collagen fibril surface potential or charge: Kelvin-probe Force Microscopy (KFM) and Fluorescence Lifetime Imaging (FLiM).

**Figure 3:**
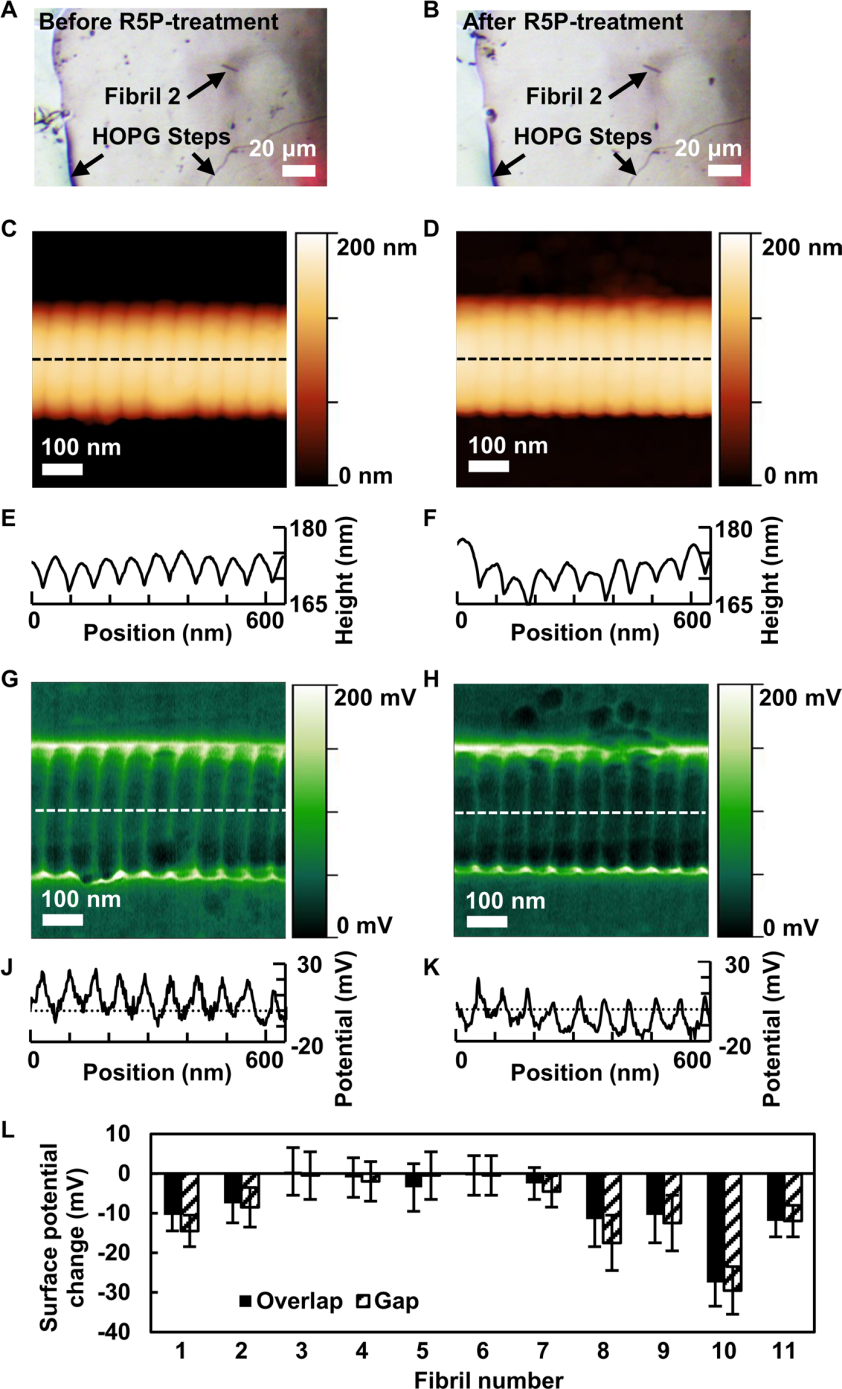
**(A,B)** Video camera images of a region of the HOPG sample with a collagen fibril before (**A**) and after (**B**) R5P treatment. **(C,D)** Representative AFM topography height images of collagen fibril number 2 from (**A,B**). Unglycated (**C**) and R5P-glycated (**D**) fibril section. **(E,F)** show longitudinal height profiles of dashed lines in (**C**,**D**), respectively. **(G,H)** Representative KFM surface potential maps of collagen fibril number 2 from (**A,B**). Unglycated (**G**) and R5P-glycated (**H**) fibril section. **(J,K)** show longitudinal potential profiles of dashed lines in (**G,H**), respectively. Surface potential values are with respect to surrounding HOPG as base level. Images and profiles (**C,E,G,J**) are on identical location of fibril. Images and profiles (**D,F,H,K**) are on identical location of fibril but different location from (**C,E,G,J**). **(L)** Effect of glycation on surface potential of overlap and gap zones of individually identified collagen fibrils (N = 11) measured by KFM. For each fibril, the overlap and gap zone surface potential reduction after R5P incubation is shown.

For the KFM assessment^48,49^, unglycated collagen fibrils were deposited on graphite (HOPG). Tapping-mode AFM topography and KFM surface potential maps were recorded on eleven fibrils before and after R5P incubation. The particular, mirror-like structure of the HOPG surface with its many, characteristically shaped step edges (Fig. 3A,B) allowed visual localisation of individual fibrils before and after R5P incubation of the whole sample using the optical camera of the AFM. While fibrils have diameters smaller than the camera resolution, their contrast against HOPG is very strong and their spatial distribution is sufficiently spread out so that individual fibrils can readily be identified. This permitted comparison of each individual fibril before and after glycation.

Exemplary topography maps of a particular fibril (fibril number 2) are shown in Fig. 3C (before R5P treatment) and Fig. 3D (after R5P treatment). The characteristic D-banding is clearly visible in both images and indicates that no significant structural or morphological change has happened. Figure 3E,F show line profiles as indicated by dashed lines in Fig. 3C,D, respectively. The average D-banding period determined by a Fast-Fourier-Transformation (FFT) analysis of the banding pattern is 65 nm ± 5 nm before glycation and 64 nm ± 5 nm after glycation, thus showing no evidence of structural alteration.

Surface charge potential maps of fibril number 2 are shown in Fig. 3G (before R5P treatment) and Fig. 3H (after R5P treatment). The D-banding is clearly visible in the surface potential before and after glycation. Higher (more positive) potential occurs in the gap zones whereas lower (more negative) potential occurs in the overlap zones. The potential contrast is not simply a topography-potential cross-talk artefact as we have shown in an earlier paper^48^. Figure 3J,K show line profiles as indicated by dashed lines in Fig. 3G,H, respectively. The potential scales (vertical axes) are with respect to the average surface potential measured on HOPG far away from the fibrils. That is, e.g., a potential of 30 mV means that this pixel has a potential 30 mV higher than that of HOPG. By comparing Fig. 3J,K, one can see that, relative to HOPG, the surface potential of the fibril is lowered after R5P incubation. This happens to, both, overlap and gap zones.

Such a comparative analysis was performed for each of the 11 fibrils. Figure 3L shows the complete set of data of the effect of R5P glycation on the surface potential for each numbered fibril individually. Glycation with R5P reduced the surface potential of the collagen fibrils, both, in gap and overlap towards more negative values in about half of all cases (fibrils 1, 2, 8, 9, 10, 11), consistent with the expected effect of glycation chemistry. In the other cases (fibrils 3, 4, 5, 6), the change was not significant. In one case (fibril 7), only the gap zone showed a significant reduction of potential. The average (N = 11) overlap potential alteration was −7.9 mV ± 8.1 mV while the average hole zone potential alteration was −9.3 mV ± 9.1 mV.

In the FLiM assessment of collagen fibril surface charge, we utilised quenching of inherent collagen fluorescence by a negatively-charged ion, triiodide (I_3_^−^). In unglycated collagen, out of the three possible fluorescent amino acids, i.e. phenylalanine, tyrosine and tryptophan, tyrosine-related emission is the main source of autofluorescence in unglycated collagen type I^50–52^, as there is no Trp in collagen type I and Phe gives only very weak fluorescence^52,53^ (see Fig. S2 for details of collagen fluorescence). Tyr is found exclusively in the collagen type I N and C-terminal telopeptides both of which are known to be oriented outwards towards the surface of collagen type I fibrils^54^; thus Tyr fluorescence is expected to be accessible to quenching by triiodide. In our R5P glycated collagen, additional fluorescence from pentosidine is present (see Fig. S2) and possibly other fluorescent crosslinks although these are expected to be in lower concentrations than pentosidine (Fig. S1)^55^. Glycation products are expected to occur preferentially on collagen fibril surfaces and can therefore be expected to be similarly sensitive to quenching by triiodide. Multi-photon excitation in the FLiM of R5P glycated and unreacted collagen allowed us to observe the collective emission from all fluorescent species – amino acids and glycation products – on the collagen fibril surfaces.

Triiodide was used as a fluorescence quencher, because it is (i) a heavy-atom quencher, which relaxes the forbidden intercombinational non-radiative and radiative conversion (S_1_→T_1_→S_0_), and thus is a very general and broad fluorescence quencher^55^; and (ii) being charged, it is a surface polarity sensor^56^. It can be expected to have access to the neutral Try, Phe and in glycated fibrils, pentosidine, fluorophores present in collagen fibrils unless the fibril surface charge is negative and repels it.

Autofluorescence decay curves were collected for R5P-glycated and control unreacted samples of collagen fibrils before and after quenching with triiodide upon broad multiphoton excitation at 750 nm and emission ≤ 495 nm, embracing all possible collagen fluorescent species, and the decay curves fitted using two exponentials (see Fig. S3 for further details).

Mean fluorescence lifetime (mean τ) was the best descriptor of changes observed in the fluorescence decay between the four experimental conditions, as it takes into account both dynamic and static quenching (Table 2). Mean fluorescence lifetime values showed that triiodide readily quenched unreacted control collagen (Tyr + Phe) autofluorescence: the mean τ suffered a drastic 10-fold decrease from 2 to 0.2 ns for control collagen fibrils upon addition of triiodide (Table 2) as expected. Meanwhile triiodide affected R5P-glycated collagen autofluorescence only slightly, reducing the fluorescence lifetime from 1 to 0.9 ns (Table 2). Thus, negatively charged triiodide ions were able to access and quench fluorescence from unreacted control collagen fibrils, but not of R5P-glycated ones. This indicates that triiodide does not get close enough to glycated collagen fibrils to have a significant effect on collagen pentosidine and amino acid fluorescence. We thus conclude that R5P glycated collagen fibrils repel the negatively-charged triiodide ions and thus that the glycated fibrils have a lower fibril surface charge compared to unreacted fibrils.

**Table 2:** Autofluorescence decay data analysis by the FLIMfit software tool^74^ glycated and unreacted control collagen before and after quenching with triiodide.

	Collagen	Collagen + I_3_^−^	Glycated collagen	Glycated collagen + I_3_^−^
**Mean τ/ns**	**1.99** ± **0.10**	**0.26** ± **0.06**	**1.09** ± **0.04**	**0.86** ± **0.04**
τ_1_/ns	5.41 ± 0.22	3.59 ± 2.92*	3.37 ± 0.25	3.7 ± 0.27
τ_2_/ps	670 ± 30	240 ± 15	510 ± 35	440 ± 25
β_1_	0.28 ± 0.01	0.01 ± 0.01	0.20 ± 0.02	0.13 ± 0.02
β_2_	0.72 ± 0.01	0.99 ± 0.01	0.80 ± 0.02	0.87 ± 0.02

Pre-exponential coefficients (β_1_ and β_2_) and fluorescence lifetime values (τ_1_ and τ_2_) are described by the decay law $f(t)={\sum }_{i=1}^{n}{\beta }_{i}{e}^{(-t/{\tau }_{i})}$. Data is presented as mean of 6–9 independent samples ± SD. Differences between τ_1_, τ_2_, β_1_, β_2_ and mean τ were found statistically significant in paired t-test for each of the four experimental conditions (except *). See also Fig. S3.

### R5P glycation reduces enzymatic intermolecular crosslinking in fibrillar collagen type I

We next assessed the extent of enzymatic crosslinking in R5P-glycated collagen fibrils compared to control, unreacted fibrils. Enzyme-mediated intermolecular collagen crosslinking is important for maintaining the molecular ordering within collagen fibrils^57^. The loss of charge ordering in collagen fibrils we observed by TEM implied that there is significant molecular rearrangement as a result of R5P-glycation. We hypothesised that for molecular rearrangement to occur in R5P glycation, there may be concomitant alterations in the intermolecular crosslinking. Recent work has demonstrated that glucose-glycated collagen fibrils contain fewer enzymatic crosslinks per mole of collagen as the extent of glycation increases^31^ and we were intrigued as to whether R5P glycation could cause a similar result. Thus, we used LC-MS analysis of the major enzymatic crosslinks to assess any effects of R5P glycation on enzymatic collagen crosslinking. The results (Table 1) showed that R5P-glycated collagen fibrils have significantly fewer HLNL cross links per mole of collagen than the control collagen fibrils (note that the analysis does not determine the distribution of crosslinks or glycation moieties amongst collagen molecules, but the bulk average per collagen molecule or mole of collagen). In addition, there are slightly fewer trivalent PYD crosslinks and more LNL and trivalent DPD crosslinks in R5P-glycated collagen fibrils implying that glycation results in an altered distribution of enzymatic crosslinks. Overall, there are significantly fewer enzymatic crosslinks per mole of collagen in the R5P glycated collagen, implying that R5P glycation can have a considerable impact on the molecular arrangement within collagen fibrils through breaking or inhibiting the formation of enzymatic crosslinks, not simply through Lys sidechain modifications.

## Discussion

The results presented above demonstrate that R5P-glycation of collagen fibrils has significant effects on the molecular arrangement within the fibrils and on the fibril surface charge. An interesting initial question is whether R5P glycation chemistry occurs only the on surfaces of collagen fibrils or throughout the fibrils. LC-MS analysis of the collagen enzymatic crosslinks showed that R5P glycation reduces the number of HLNL crosslinks, the dominant enzymatic crosslink present, from approximately one per collagen molecule (consistent with the maximum possible number of crosslinking sites per collagen molecule being four) to less than one per two collagen molecules. Such a drastic reduction in the number of HLNL crosslinks strongly suggests glycation chemistry occurs throughout the fibril and not just on surface molecules. The number of surface molecules on a circular cross-section fibril is proportional to R/r where R is the radius of the fibril, here 50–200 nm, and r is the radius of a collagen molecule, of order 1 nm. In comparison, the number of molecules in the fibril as a whole is governed by (R/r)^2^, and thus here is of order two orders of magnitude larger than the number of molecules on the fibril surface. That HLNL crosslinks amount to roughly one per collagen molecule out of a maximum possible of four per collagen molecule indicates that HLNL crosslinks must occur throughout the fibril and not just on the fibril surface; if HLNL crosslinks were present only on surface collagen molecules, the total number of HLNL crosslinks per collagen molecule would be significantly less than one. If R5P glycation affects only surface HLNL crosslinks, only ~1/100 of the HLNL crosslinks would be affected, and thus only a minimal reduction in the overall number of HLNL crosslinks per collagen molecule in the fibril would be observed. That R5P glycation reduces the number of HLNL crosslinks per collagen molecule to less than a half suggests that the R5P effect on HLNL crosslinks must be occurring throughout the fibril and not just on the surface molecules and thus that glycation chemistry is likely to be occurring throughout the fibril structure.

### R5P glycation has a significant effect on the arrangement of molecules in collagen fibrils

TEM images of negatively-stained R5P-glycated collagen fibrils showed that R5P-glycated collagen fibrils have a significantly broader distribution of D-period lengths than unreacted fibrils, consistent with findings from diabetic rat tendon and glucose-glycated collagen^58^, and images of both positively and negatively-stained fibrils exhibited broadening of the sub-band staining. Together, these observations are consistent with the disordering of collagen molecule positions in R5P-glycated fibrils. The nature of the disorder could be in the longitudinal arrangement of the collagen molecules within the fibril structure and/or in the tilt angle between collagen molecules or molecular segments and the fibril longitudinal axis^57,59^; both possibilities are consistent with our TEM observations. The 234-amino acid residue D periodicity in the collagen molecular arrangement is widely believed to be driven by intermolecular charge-charge interactions between sidechain termini on neighbouring molecules^45–47^. R5P-glycated Lys/Hyl residue sidechains terminate in neutral (sugar adducts, N-acetyl) or negatively-charged (CML, CEL) functionalities (see Fig. S1), rather than the positively-charged terminal amine group of unglycated residues. Thus, stabilising positive-negative intermolecular charge pair interactions in unglycated collagen become either neutral-negative or repulsive negative-negative pairs for glycated Lys residues, and thus contribute destabilising rather than stabilising interactions in the collagen fibril. Reduction in the number of the charge-charge interactions that stabilise the longitudinal arrangement of collagen molecules in the fibril structure may be an underlying cause of the observed disordering in R5P-glycated collagen fibrils. If this is indeed the case, then it might also be expected that adhesion between collagen molecules is weaker in glycated fibrils and therefore that the fibrils are more easily disrupted, e.g. by migrating cells. Previous work on tendons from diabetic patients^60^ has shown a range of morphological abnormalities in collagen fibrils, hypothesised to come from glycation over many years, presumably in tandem with repetitive mechanical strains on the fibrils, suggesting that glucose-glycated collagen fibrils are more readily morphologically disrupted by mechanical force than normal collagen fibrils. Additionally, the longer sidechains on glycated residues may also contribute to molecular disordering in the fibril structure by inhibiting close packing of collagen molecules, and thus reducing the strength of the remaining charge-charge and hydrophobic interactions. Interestingly, our LC-MS measurements showed that R5P glycated type I collagen fibrils have fewer enzymatic crosslinks than unreacted fibrils. Enzymatic crosslinks between N and C-terminal telopeptides not only regulate collagen fibril stiffness, they are significant in maintaining molecular ordering within the fibrils. Reducing the number of enzymatic crosslinks thus can be expected to facilitate molecular reorganization within the fibrils. AGE crosslinks are the endstage products of multistep reaction processes and so likely only form once a significant degree of molecular reorganisation has already occurred. We speculate that AGE crosslinks may “cement” in place the new molecular alignments that occur as a result of glycation-altered charges and sidechain bulkiness.

### The changes to molecular alignment with glycation of collagen fibrils can be expected to affect cell adhesion and migration

We have previously shown that (Gly-Pro-Hyp triplets) align in the fibril structure and that the integrin (cell adhesion) binding sites on collagen fibrils are adjacent to bands of Gly-Pro-Hyp triplets^61^. The Gly-Pro-Hyp triplets have well-defined flexibility^61^ and their alignment in the fibril structure confers the molecular flexibility locally across the fibril. Disordering of the molecular alignment of collagen molecules as we have observed by TEM will affect the alignment of the Gly-Pro-Hyp triplets, which in turn can be expected to affect the local fibril flexibility around integrin binding sites and disrupt the collagen-integrin binding dynamics with consequential impact on cell adhesion and migration.

### The addition of chemical groups to collagen molecules and the alteration in collagen fibril surface charge as a result of R5P glycation will impact on the binding of other ECM components to glycated collagen fibrils

The collagen fibril sub-bands most strongly affected by R5P glycation in TEM images are those in the gap zone and interface between gap and overlap zones where collagen molecule C-terminal telopeptides are situated. These same fibril regions contain the binding sites for important ECM components: fibronectin and the proteoglycans that interact with collagen, e.g. decorin^62^ and biglycan^63,64^. Glycation in these collagen fibril regions will impact the non-covalent interactions that mediate binding of proteoglycans and fibronectin to collagen fibrils and so may have consequences for the structural integrity of the ECM around R5P-glycated collagen fibrils. That the collagen fibril surface charge reduces with R5P glycation, more so in the fibril gap zone, is also relevant for binding between collagen fibrils and surrounding ECM molecules, particularly for the proteoglycans because of their negatively-charged glycosaminoglycan chains. It has previously been shown that acid-soluble (i.e. non-crosslinked), glucose-glycated collagen molecules have lower affinities for heparin and keratan sulfate proteoglycans than their unglycated counterparts^39^. If a similar trend occurs *in vivo* between collagen fibrils and surrounding proteoglycans, we would surmise that collagen fibrils can be more easily separated from the surrounding proteoglycan hydrogel in ECM subject to R5P glycation. Such a feature is potentially highly relevant for migrating cancer cells that need to separate ECM molecules as they advance^7,65^.

The effects of R5P glycation can be expected to be especially relevant in the environment of actively-dividing cells, for example, during embryo attachment in mammals^66^, when the blastocyst interacts with endometrial epithelial cells^67^ and during the migration and attachment of cancer cell during metastasis^68,69^. Our results here show that there can be significant changes in the physicochemical properties of collagen fibrils from R5P-collagen glycation that will impact on integrin-mediated cell adhesion and migration and on the adhesion of surrounding ECM molecules, which additionally plays a role in cell migration. These results suggest a significant potential for R5P glycation to influence the cellular microenvironment in cancer especially, where cell necrosis gives a direct route to high R5P extracellular concentrations. Urgent further research is needed to understand the impact of this glycation potential on cell signalling, adhesion and migration in the cancer setting.

## Methods

All reagents were from Sigma-Aldrich except (U-^13^C_5_)-ribose-5-phosphate the synthesis of which is described in Li *et al*.^38^.

### Glycation of collagen

Bovine Achilles tendon type-1-collagen fibrils (Sigma-Aldrich Company Ltd., Dorset, United Kingdom) were dispersed either in PBS or in 50 mM ribose-5-phosphate solution (U-^13^C-R5P for the NMR experiments in Fig. S1, unlabelled R5P for all other experiments) in PBS containing 0.01% sodium azide, pH 7.4. Samples were left to incubate at 37 °C for 5 weeks. After incubation, both control and glycated collagen were washed several times with deionised water to remove unreacted sugar, phosphate, and soluble glycation products and used for further analysis. ^13^C-Ribose and glucose-glycated samples for NMR spectroscopy (Fig. S1) were similarly prepared using the appropriate U-^13^C-sugar (Cambridge Isotope Laboratories, USA) except that the glucose-glycated sample was left to incubate for 12 weeks, because of the slower reaction time for glucose. For the Cu^2+^-edited NMR spectrum of glucose-glycated collagen, used to identify the NMR signals from the Amadori glycation products, a sample of the prepared U-^13^C-glucose-collagen glycation material was left in a solution of D_2_O and CuCl_2_. 2H_2_O (0.1 M) for 1 hour, sonicated for 30 seconds and then left to stand for a further one hour. The sample was then centrifuged at 7000 rpm for 3 minutes and the upper layer of liquid removed. Fresh D_2_O was added and the mixture shaken. This centrifuging washing routine was repeated twice to remove all non-bound Cu^2+^.

### Preparation of collagen/glycated collagen suspensions for TEM, AFM, KFM and FLiM

10 mg/ml Bovine Achilles tendon collagen or glycated collagen was suspended in PBS and sonicated for 10 mins (cycle-30 sec on, 30 sec off, amplitude 40%) so that the collagen was dispersed in the suspension.

#### Mass spectroscopy

*Acid hydrolysis*. Collagen (dry weight 4 mg) suspended in 200 μl PBS pH 7.4 was reduced by the addition of 10 μl of 10 mg/ml NaBH_4_ in 1 mM NaOH. After incubation for 2 h at room temperature the sample was washed 3 times with water and then freeze dried. Acid hydrolysis was carried out by incubation overnight at 95 °C with 200 μl of 7.4 M HCl. Samples were then dried under a stream of nitrogen gas and dissolved in 400 μl 30% MeCN/0.1% formic acid. After filtering through a 0.22 μm nylon filtration membrane samples were freeze dried before resuspension at 10 μg/μl (dry weight) in 30% MeCN/0.1% formic acid. Solutions were then made up at different concentrations with appropriate internal standards for the analysis of the different analytes.

### HPLC-MS method

5ul of solution for analysis was injected onto a Cogent Diamond Hydride column (4 μm, 100 A, 150 ×2.1 mm). Diamond Hydride columns have been described in the analysis of amino acids^70^ and collagen crosslinks^71^. The method here is a modification of these protocols. A gradient of 100% (acetonitrile, 5% water, 0.1% formic acid, 0.005% trifluoroacetic acid) to 100% (water, 0.1% formic acid) was run, the details of which are in Table 3 below. The flow was passed into an esi probe of a Micromass Quattro Ultima mass spectrometer and the fragmentation transitions listed below (Table 4), monitored. (Mass spectrometer parameters: source temperature 120 °C, desolvation temperature 350 °C, cone voltage 3 kV, capillary voltage 35 V, collision gas was argon, collision voltage - see Table 4).

**Table 3:** HPLC gradient table.

Time/min	% solvent A	% Solvent B	% Solvent C	Flow/ml/min	Curve
0	100	0	0	0.4	1
5	60	40	0	0.4	6
7	10	90	0	0.4	6
9	0	0	100	0.4	1
11	0	0	100	0.4	1
12	100	0	0	0.4	1
20	100	0	0	0.4	1

System: Waters Alliance 2795.

Solvent A: (95% acetonitrile, 5% water) 0.1% formic acid, 0.005% Trifluoroacetic acid.

Solvent B: (20% methanol, 80% water), 0.1% formic acid.

Solvent C: water, 0.1% formic acid.

Curve 6 is a linear gradient, Curve 1 is a step change to the indicated percentage solvent.

**Table 4:** MRM transitions and collision energy settings for collagen amino acids and crosslink components.

Molecule	Q1	Q2	Collision energy	dwell/s
Proline (PRO)	116.07	70.06	15	0.25
d7-PRO	123.11	77.11	15	0.25
Hydroxyproline (HYP)	132.06	68.05	20	0.25
Lysine (Lys)	147.11	84.08	20	0.25
d4-LYS	151.14	88.1	20	0.25
Hydroxylysine (Hly)	163.1	82.06	20	0.25
LNL	276.15	84.08	30	0.2
HLNL	292.18	82.08	30	0.2
DHLNL	308.18	82.08	35	0.2
pentosidine	379.21	187.1	40	0.2
deoxypyridinoline	413.2	84.08	40	0.3
pyridinoline	429.2	82.08	40	0.3

### Calibrations

Amino acid calibration curves were made using a commercially available amino acid mix from Sigma Aldrich (A9906 lot SLBR9938V). Calibration curves were constructed using 2,3,3,4,4,5,5-d7- DL-Pro (CK Isotopes) as internal standard for Pro, Hyp and 4,4,5,5-d4-L-Lysine as internal standard for Lys and Hly.

HLNL, LNL and DHLNL (Santa Cruz Biotechnology Inc.) were used for calibration curves with d4-lysine (Sigma Aldrich) as an internal standard. The standards and ISD were spiked into acid hydrolysed collagen (50ug) which had not been reduced for the calibration curve.

DPD (Polypeptide Group) was used to construct a calibration curve against d4 Lysine as the internal standard. Calibration curves we constructed separately and confirmed for DPD and PYD with a PYD/ DPD HPLC mixture (Quidel Corporation).

#### NMR spectroscopy (see SI)

All solid-state NMR spectroscopy was performed on a Bruker AVANCE II 400 MHz instrument using standard Bruker double resonance probes and 4 mm zirconia rotors. All collagen and glycated collagen samples were freeze-dried prior to NMR spectroscopy.

^13^C{^1^H} cross polarization was performed on control and glycated samples with 10 kHz magic angle spinning, 2.5 ms contact time (ramped ^1^H, square ^13^C contact pulses), ^1^H 90° pulse length 2.5 μs, broadband ^1^H decoupling (nutation field 100 kHz) during acquisition, recycle delay 2 s, and external chemical shift referencing on the methylene ^13^C signal of glycine at 43.1 ppm relative to TMS at 0 ppm.

The 1D SQ-DQ-filtered ^13^C NMR spectra used 10 kHz magic-angle spinning and the POST-C7 pulse sequence^72^ with a 70 kHz ^13^C nutation field (3.57 μs pulse length) to excite double quantum coherence in 0.4 ms. Magnetisation was returned to zero quantum by another 0.4 ms of POST-C7 sequence. During double quantum evolution, 100 kHz Lee-Goldberg decoupling was applied on ^1^H. The 2D ^13^C proton-driven spin-diffusion experiments^73^ used CP parameters as above. The ^13^C transverse magnetisation was allowed during the *t*_1_ incremental delay and returned to zero quantum coherence by a ^13^C 90° pulse (3.57 μs). ^1^H decoupling was switched off during this mixing period (100 ms), with a ^13^C 90° readout pulse at the end of the mixing period. During both the incremented delay and acquisition periods, SPINAL64 decoupling was applied on ^1^H at 100 kHz nutation frequency.

^31^P {^1^H} cross polarization NMR spectroscopy was performed on R5P (Sigma-Aldrich) and freeze-dried glycated collagen samples with 10 kHz magic angle spinning, contact time 10 ms, ^1^H 90° pulse length 2.5 μs, broadband TPPM decoupling (nutation field 100 kHz) during acquisition, recycle delay 2 s and were referenced to crystalline hydroxyapatite at 2.8 ppm relative to 85 wt% H_3_PO_4_ at 0 ppm.

#### Bright Field Transmission Electron Microscopy (BF-TEM)

5 μl of unglycated and R5P-glycated collagen suspension was adsorbed onto glow-discharged 400 mesh copper/carbon-film grids (EM Resolutions) for about 2 min. Grids were rinsed on two drops of DIW and positive/ negative staining was performed using a 2% aqueous uranyl acetate solution. For positive staining, the grid was passed again over two drops of DIW after uranyl acetate staining to remove excess stain and the grid allowed to dry before transferring to the TEM.

Grids were viewed in an FEI Tecnai G^2^ electron microscope run at 200 keV using a 10 μm objective aperture. Images were acquired using AMT Camera software. The plot profiles of images were analysed using Image J software after rotating images to generate horizontal alignment of fibrils to be analysed. For analysis of the D-period length and SD, 15 measurements on 5 unreacted collagen type I fibrils were made, i.e. 3 measurements per fibril, and 12 measurements on 4 glycated fibrils (images shown in Fig. 2E), i.e. 3 measurements per fibril. Standard deviations for D-periods measured on a single fibril were very close to the image pixel size (0.4–0.5 nm) in all cases, i.e. SD of intrafibril variation of the D period is essentially at the level of image resolution, and thus intrafibril variability was considered to be negligible.

#### AFM, KFM

The methodology used was similar to that used previously^49^. Small drops of fibril suspension (ca. 50 μl) were put onto freshly cleaved pieces of HOPG (Fig. 3) (ZYB-grade, Bruker Corporation, Billerica MA, USA), left for ca. 10–20 min. and then briefly rinsed with DIW. The water was then blown off with ambient air using manually-operated bellows. AFM/KFM was performed with a Dimension Icon FastScan AFM (Fig. 3) (Bruker Corporation, Billerica MA, USA) with Nanoscope V controller in ambient air (relative humidity = 20% –30%, temperature = 21 °C –25 °C). Bruker TAP150A, n-doped (Sb) Si-tips (nominal cantilever spring constant = 5 N/m, nominal tip radius = 10 nm) were used for all AFM/KFM measurements. The same individual tip was used for all measurements.

All KFM measurements (Fig. 3) were performed in the usual two-pass mode, where each scan line is traced twice, first to record the topography in tapping mode, then to record the surface potential by AM-KFM with the tip retracing the topography while maintaining a constant tip-sample distance. Nominal lift height was zero in all measurements.

An external control system was used for the AM-KFM operation in order to increase sensitivity. This system consisted of an off-the-shelf function generator (InfiniiVision DSO-X 2004A, Keysight, Santa Rosa CA, USA), which applied a sinusoidal signal with the cantilever’s first resonance frequency to the tip *via* the *tip bias* input of the Bruker Signal Access Module (SAM). The resulting deflection signal of the tip oscillation was taken from the SAM and fed into an external lock-in amplifier (7270 DSP, Ametek Inc, Berwyn PA, USA) to determine the in-phase amplitude component of the oscillation (in-phase with the signal from the function generator). This signal is then the error signal of the control circuit. A custom-made analogue-controller was used to perform the standard AM-KFM control procedure and the control signal, which represents the actual surface potential to be determined, was fed back to the tip *via* the SAM. The control signal was also input to the customisable *Input1* port of the AFM, where it was digitized by the Nanoscope V controller and its data acquisition system for image analysis^49^.

All image data was analysed using the free, third-party data analysis software Gwyddion (gwyddion.net). The D-banding period of the fibril topography was determined by cropping out a 16-pixel wide and approximately 600 nm long area in the center of a fibril and applying the *2D-FFT* function to it. Clear peaks were found around 15 µm^−1^ in the fourier-transformed images, from which the banding-periods could be calculated by inverting the values. The fibril potentials (Fig. 3L) were determined as follows: the potential map was oriented such that a single fibril appeared vertical and the image was 1^st^-order line-levelled with the *align rows* function excluding the fibril itself using the *mask* function. Then, a 16-pixel (= 20 nm) wide and 500-pixel (= 1000 nm) long, rectangular area in the center of the fibril was cropped out. On this area, the *mark grains by threshold* function with 50% threshold level was used to identify all pixels above and below the threshold, respectively. Of these pixels, the average potential values of overlap zone and gap zone, respectively, were calculated using the *statistical quantities* function. The actual locations of the zones were determined from the topography maps.

#### FLiM

Fluorescence lifetime measurements were obtained on upright multi-photon scanning fluorescence microscope, (LaVision BioTec TriM Scope II (LaVision BioTec GmbH, Bielefeld, Germany) equipped with Insight Deepsee laser light source (the Spectra-Physics, Santa Clara, CA, USA). The measured fluorescence decay curves were fitted using two exponentials using the FLIMfit software tool developed at Imperial College London^74^. The calculated lifetimes (τ_1_ and τ_2_) of our samples (Table 2) were in good agreement with those reported previously^75,76^.

#### Fluorescence spectroscopy (see SI)

Fluorescence excitation and emission spectra were obtained on Cary Eclipse fluorescence spectrophotometer (Agilent, former Varian, Santa Clara, CA, USA). Quartz SUPRASIL® 10 ×2 mm high precision cells (Hellma Analytics, Hellma GmbH & Co, Mullheim, Germany) were used. Because of the weak fluorescence, the band widths of both excitation and emission monochromators were set at 5 nm. Fluorescence emission spectra were recorded manually at excitation wavelengths 265–375 nm with 10 nm interval. Fluorescence excitation spectra were recorded for every maximum/shoulder in the emission spectra. All spectra were corrected for instrumental distortions.

## Supplementary information

Supplementary information

## Data Availability

Raw data can be obtained from Melinda J Duer, email: mjd13@cam.ac.uk.
