# Supplementary material for: Glycation changes molecular organization and charge distribution in type I collagen fibrils
Source: Sci Rep. 2020 Feb 25;10:3397. doi: 10.1038/s41598-020-60250-9 (PMC7042214; doi:10.1038/s41598-020-60250-9)
Supplement: Supplementary file 1 — Supplementary information [file 41598_2020_60250_MOESM1_ESM.pdf]

Glycation changes molecular organization and charge distribution in type I collagen fibrils

Sneha Bansode, Uliana Bashtanova, Rui Li, Jonathan Clark, Karin H. Müller, Anna Puszkarska, Ieva Goldberga, Holly H. Chetwood, David G. Reid, Lucy J. Colwell, Jeremy N. Skepper, Catherine M. Shanahan, Georg Schitter, Patrick Mesquida, Melinda J. Duer**

Supplementary Information

NMR spectroscopy and LC/MS characterisation of R5P-collagen glycation products

Solid-state NMR spectroscopy assessment of the R5P-glycation products of collagen type I (bovine tendon) fibrils is fully described in references 29 and 30 of the main text. Fig S1 summarises the solid-state NMR data for the samples used here and the assignment of NMR signals to glycation products. Spectra for collagen type I glycated with U-^{13}C glucose and U-^{13}C-ribose whose glycation chemistries are more extensively described in the literature are also given for comparison. Pentosidine (the major fluorescent glycation product expected) signals cannot be distinguished from those from other unsaturated glycation products but was quantified by LC-MS (see Materials and methods for details) to be present at 0.03 ± 0.01 pentosidine crosslinks per collagen molecule. We did not quantify other glycation products by LC-MS as to do so requires at least one model compound for each possible glycation product and the very large number of possible glycation products in the case of R5P glycation (see NMR assessment and references 29 and 30) is prohibitive for this strategy.

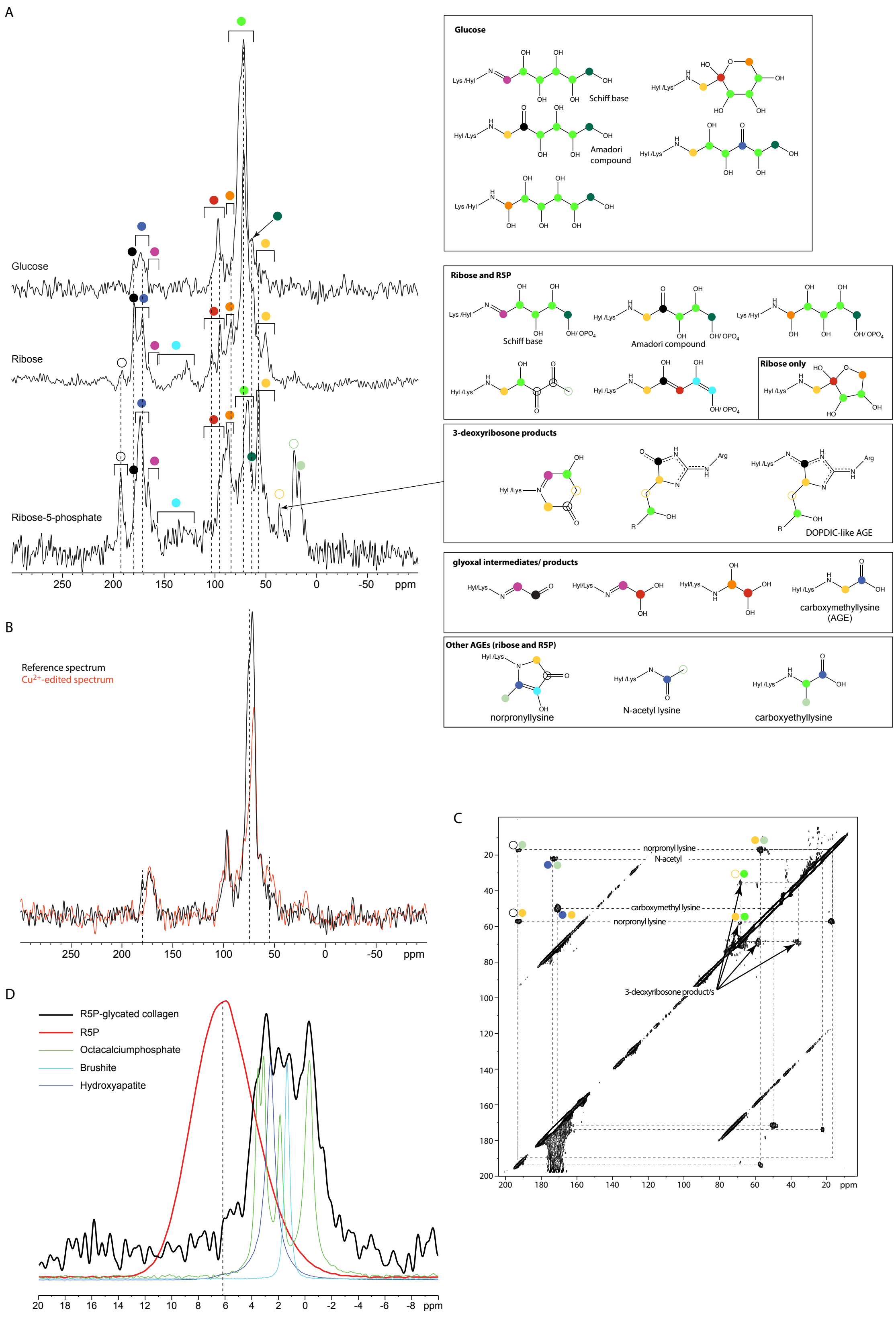
A
Glucose
Ribose
Ribose-5-phosphate
250 200 150 100 50 0 -50 ppm
Glucose
Lys /Hyl
Schiff base
Hyl /Lys
Amadori compound
Ribose and R5P
Schiff base
Amadori compound
OH/ OPO4
Ribose only
3-deoxyribosone products
Arg
DOPDIC-like AGE
glyoxal intermediates/ products
carboxymethyllysine (AGE)
Other AGEs (ribose and R5P)
norpronyllysine
N-acetyl lysine
carboxyethyllysine
B
Reference spectrum
Cu2+-edited spectrum
250 200 150 100 50 0 -50 ppm
C
norpronyl lysine
N-acetyl
carboxymethyl lysine
norpronyl lysine
3-deoxyribosone product/s
200 180 160 140 120 100 80 60 40 20 ppm
D
R5P-glycated collagen
R5P
Octacalciumphosphate
Brushite
Hydroxyapatite
20 18 16 14 12 10 8 6 4 2 0 -2 -4 -6 -8 ppm

Fig S1: (A) Left: 1D [1]double-quantum-filtered solid-state ^{13}C NMR spectra of bovine collagen type I fibrils glycated with U-^{13}C-glucose, U-^{13}C-ribose and U-^{13}C-R5P. The spectra contain only signals from ^{13}C bonded to another ^{13}C and so contain only signals from molecular species derived from the respective ^{13}C-labelled sugar. Possible assignments for each signal/ range of signals are indicated with coloured circles; the similarity of chemical structure between possible glycation products means that there is both overlap of signals from different glycation products and several possible assignments for most signals. Dotted lines are to allow easy comparison of chemical shifts of signals for the different glycation sugars. Right: possible glycation products with ^{13}C site chemical shifts expected indicated with coloured circles that correspond to the colour scheme used to designate signals in the spectra on the left. (B) 1D double-quantum-filtered solid-state ^{13}C NMR spectra of bovine collagen type I fibrils glycated with U-^{13}C-glucose with (red) and without (black) addition and short incubation of aqueous Cu^{2+} with the U-^{13}C-glucose-glycated sample. Cu^{2+} preferentially coordinates to Schiff bases and Amadori products and, being a paramagnetic ion, has the effect of reducing signal intensity from the ^{13}C in these molecular species (affected signals indicated with dotted lines) allowing assignment of the NMR signals from these molecular species (as shown in part (A), top). (C) 2D ^{13}C-^{13}C proton-driven spin-diffusion correlation spectrum for U-^{13}C-R5P-glycated collagen type I, showing the assignment of norpronyl-lysine, (carboxymethyl)lysine, N-acetyl and products of Lys-Arg glycation with 3-deoxyribosone-like molecules as the major collagen - R5P glycation products. Products of 3-deoxyribosone-like molecules are expected to include products, such as DOPDIC, with a -CH(N)….CH_2-CH(OH)- fragment (*1*). The $^{13}CH_2$ component in such fragments has a distinctive ~37 ppm signal (*1*). (D) ^{31}P cross-polarization solid-state NMR spectrum of lyophilized U-^{13}C-R5P used as the starting material in the R5P glycations in this work (red) and of the final R5P-glycated type I collagen (black). The spectrum of the final glycated material (black) consists of overlapped spectra from various inorganic calcium phosphates (octacalcium phosphate, hydroxyapatite and brushite), indicating

that most of the original R5P organic phosphate has been released as inorganic phosphate which precipitates with residual calcium ions (presumably bound originally to the collagen fibrils).

Fluorescence spectral analysis of glycated collagen

Tyrosine exhibits well-known excitation/emission maxima at 274/303 nm (*2*). Raw fluorescence spectra of unglycated collagen showed the expected tyrosine emission maximum at 303 nm when excited at 275 nm (Fig. S2A). Difference fluorescence spectra obtained by subtraction of emission of glycated collagen from unglycated (Fig. S2B) showed that other fluorescence species in the unglycated collagen were also present and their fluorescence was also quenched during glycation. These species can be attributed to the tyrosine oxidation events in tendon tissue: DOPA (285/312 nm) and its oxidation products (a broad shoulder at 320-400 nm) (*3*, *4*). The presence of DOPA and its oxidation products was also obvious from the broadening of the emission spectrum to a half-height peak linewidth of 60 nm (Fig. S2B) instead of 35 nm expected for tyrosine alone.

The fluorescence spectra of collagen after glycation with R5P (Fig S2A) showed a considerable decrease in tyrosine-related fluorescence, that could be due to one or a combination of several possible chemical events resulting in tyrosine quenching: (i) glycation of amino acids in the vicinity of tyrosine (ii) changes in the collagen fibril molecular organisation to bring quenching glycated residues into close proximity to N and C-terminal telopeptides, , or (iii) glycation of tyrosine itself (Tyr is not expected to react directly with R5P, but glycation reactions on nearby Lys or Arg residues may result in activation of Tyr and subsequent glycation or oxidation reactions, or oxidization by glycation by-products) .

A

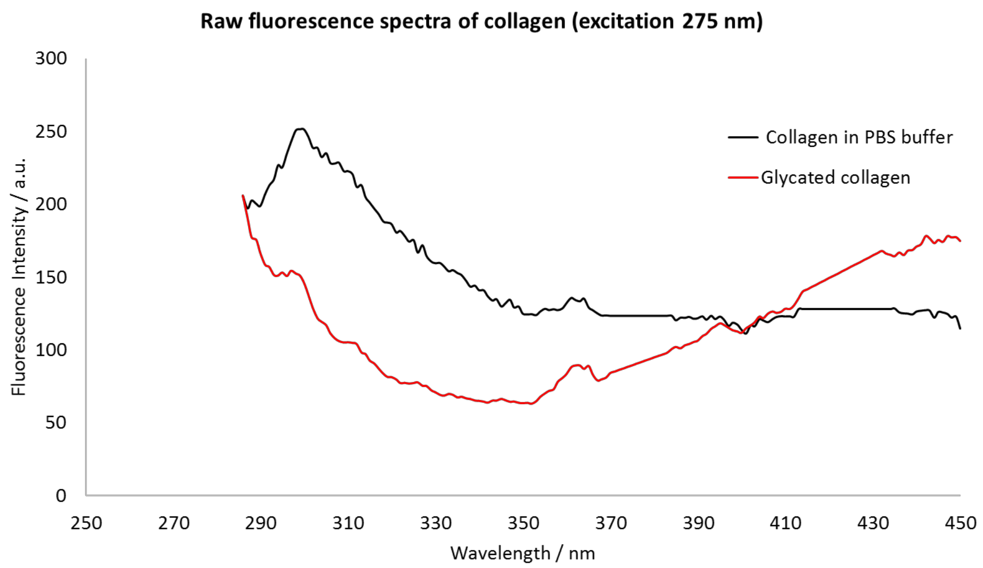
Raw fluorescence spectra of collagen (excitation 275 nm)
Fluorescence Intensity / a.u.
Wavelength / nm
Collagen in PBS buffer
Glycated collagen
0
50
100
150
200
250
300
250
270
290
310
330
350
370
390
410
430
450

B

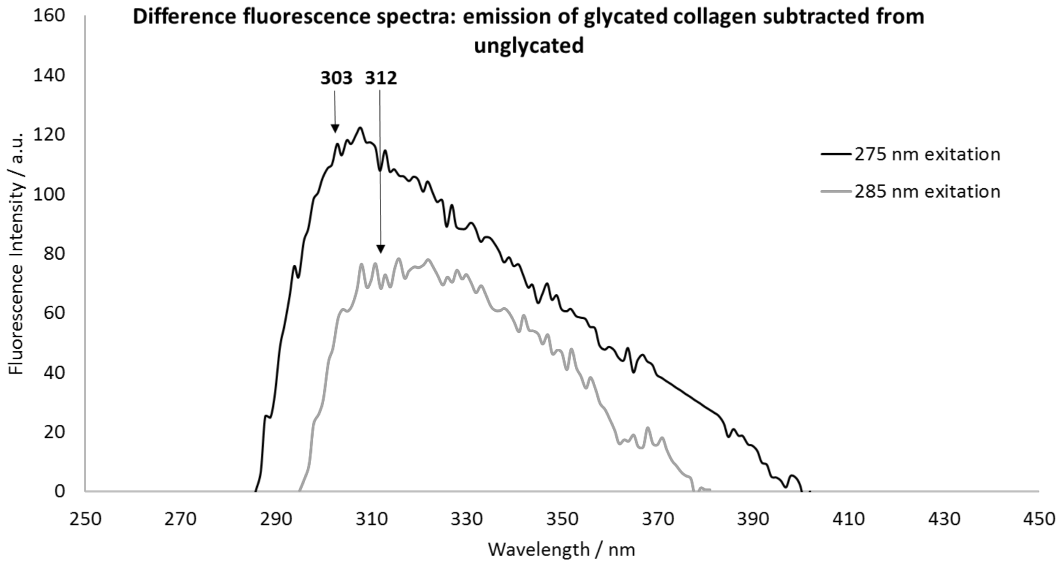
Difference fluorescence spectra: emission of glycated collagen subtracted from unglycated
303
312
275 nm exitation
285 nm exitation
Fluorescence Intensity / a.u.
Wavelength / nm
0
20
40
60
80
100
120
140
160
250
270
290
310
330
350
370
390
410
430
450

C

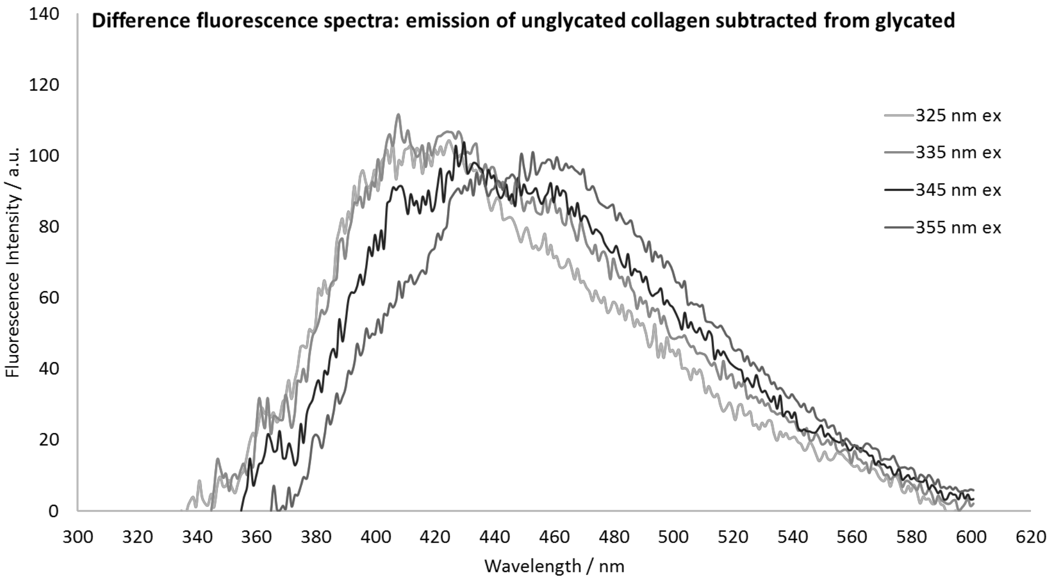

Fig S2: Effect of R5P-glycation on fluorescence spectra of collagen fibrils. **(A)** Raw spectra show decrease of amino acid (Tyr) emission in collagen after glycation with R5P (excitation 275nm). **(B)** Difference emission spectra when excited with 275 and 285 nm. **(C)** Difference emission spectra of AGE products in R5P-glycated collagen fibrils (excitation 325-355 nm).

Collagen glycation results in formation of advanced glycation end products (AGEs), some of which give significant fluorescence intensity even at low abundance, e.g. pentosidine (*5*). AGE fluorescence is expected at higher excitation and emission wavelengths than Tyr, and are thus easily distinguishable (*2*, *6*). On 325 nm excitation, R5P-glycated collagen showed a broad and unstructured fluorescence increase, while no fluorescence was associated with unglycated collagen. Difference fluorescence spectra (Fig. S2C) obtained by subtraction of emission of unglycated from glycated collagen over an excitation range of 325-355 nm showed that emission wavelength maximum and half-height linewidth both change with excitation, indicating the presence of several fluorescent AGE species, consistent with our LC/MS analysis which identified the presence of pentosidine and solid-state NMR analysis (Fig S1) which identified several deoxyribusone products some of which may show fluorescence. The 300-325/ 400-430 nm fluorescence also can be attributed to dityrosine,

which is formed from neighbouring tyrosine residues and associated with cross-linking in biological materials including collagen (*3*, *4*).

Fluorescence lifetime imaging (FLiM) of glycated collagen

Autofluorescence decay curves (Fig S3) were collected for R5P-glycated and unglycated samples of collagen fibrils before and after quenching with triiodide upon broad multiphoton excitation at 750 nm and emission ≤ 495 nm to embrace all feasible collagen fluorescent species identified by steady-state fluorescence spectroscopy (see Fig S2 and related text) and the decay curves fitted using two exponentials (for more details see Table 1 in the main text).

A

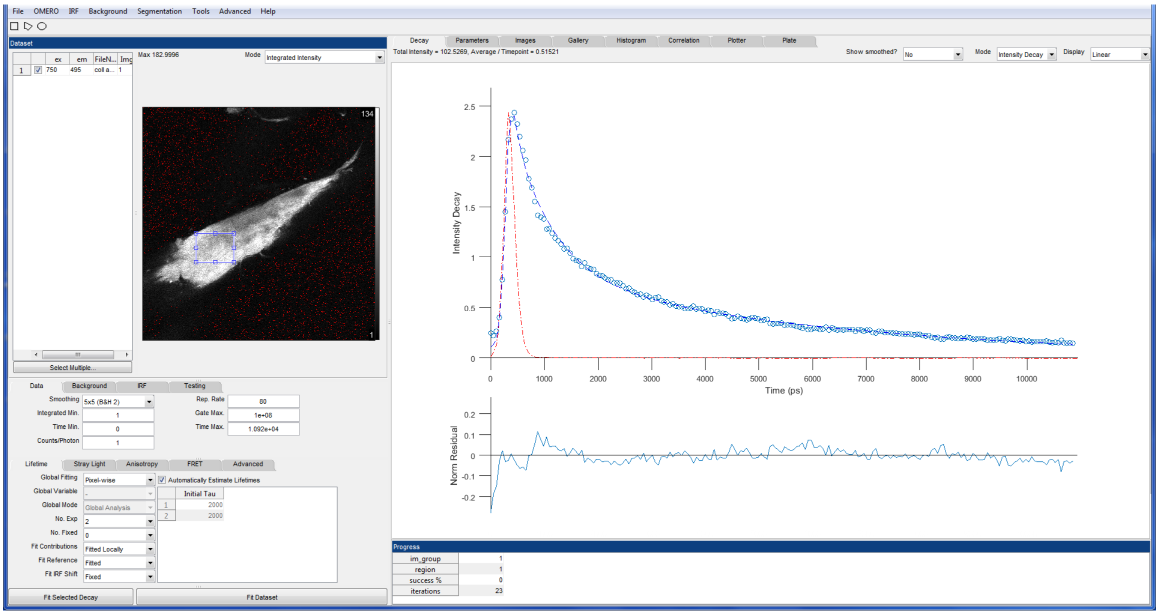

B

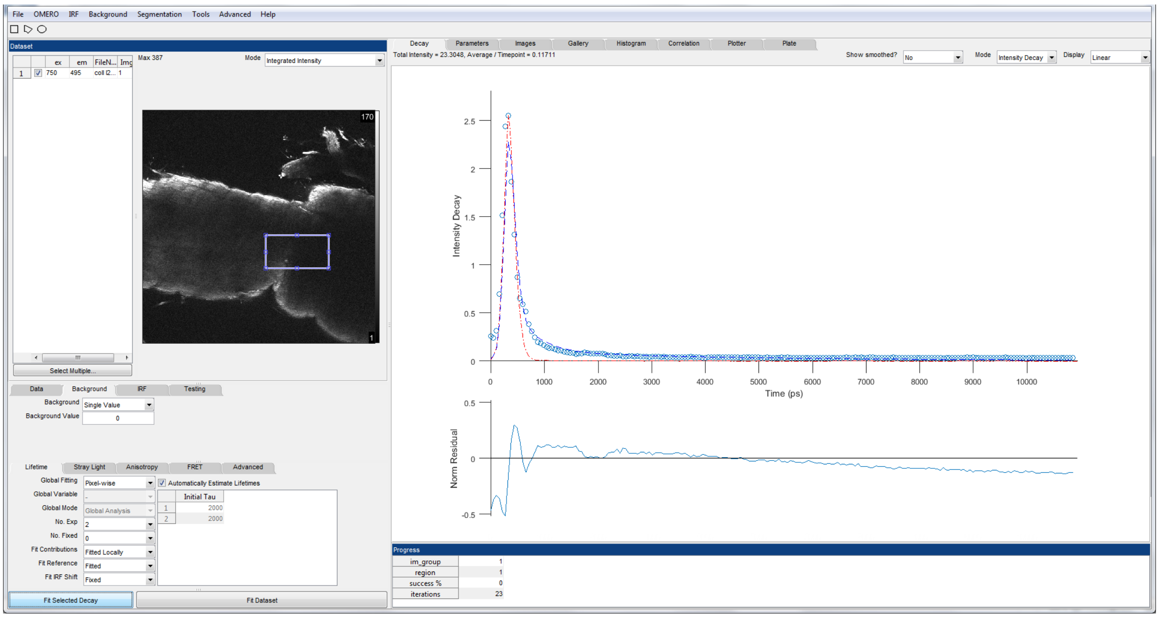
File OMERO IRF Background Segmentation Tools Advanced Help
Dataset
Max 387
Mode Integrated Intensity
Decay Parameters Images Gallery Histogram Correlation Plotter Plate
Total Intensity = 23.3048, Average / Timepoint = 0.11711
Show smoothed? No
Mode Intensity Decay
Display Linear
Intensity Decay
Time (ps)
Norm Residual
Select Multiple...
Data Background IRF Testing
Background Single Value
Background Value 0
Lifetime Stray Light Anisotropy FRET Advanced
Global Fitting Pixel-wise
Automatically Estimate Lifetimes
Global Variable
Global Mode Global Analysis
No. Exp 2
No. Fixed 0
Fit Contributions Fitted Locally
Fit Reference Fitted
Fit IRF Shift Fixed
Initial Tau
2000
2000
Progress
im_group 1
region 1
success % 0
iterations 23
Fit Selected Decay
Fit Dataset

C

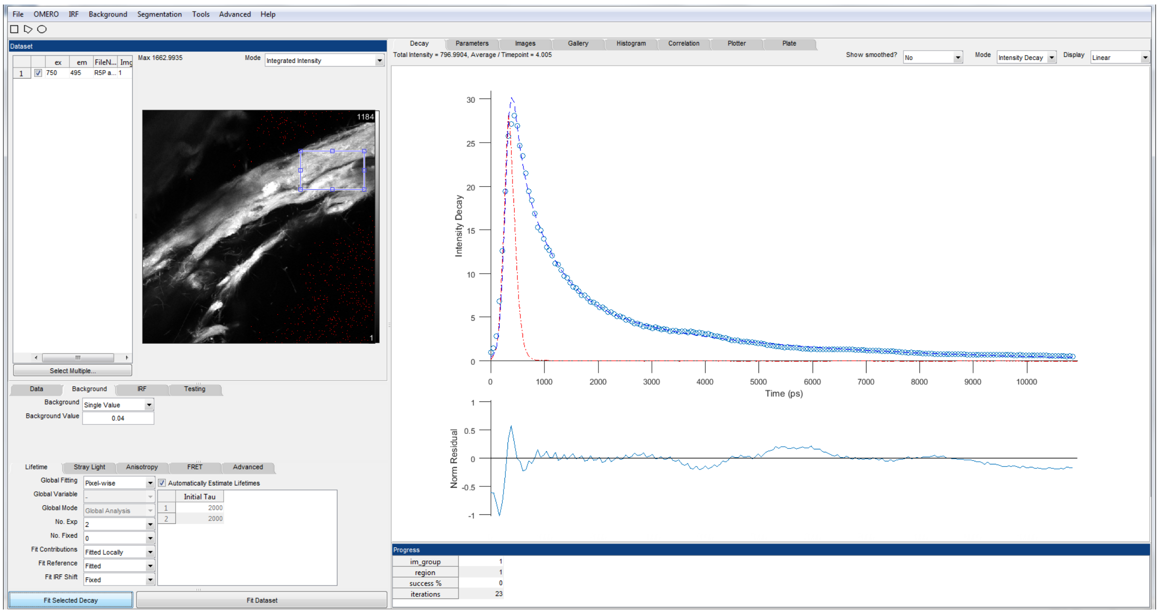
File OMERO IRF Background Segmentation Tools Advanced Help
Dataset
Max 1662.9935
Mode Integrated Intensity
Decay Parameters Images Gallery Histogram Correlation Plotter Plate
Total Intensity = 796.9904, Average / Timepoint = 4.005
Show smoothed? No
Mode Intensity Decay
Display Linear
Intensity Decay
Time (ps)
Norm Residual
Select Multiple...
Data Background IRF Testing
Background Single Value
Background Value 0.04
Lifetime Stray Light Anisotropy FRET Advanced
Global Fitting Pixel-wise
Automatically Estimate Lifetimes
Global Variable
Global Mode Global Analysis
No. Exp 2
No. Fixed 0
Fit Contributions Fitted Locally
Fit Reference Fitted
Fit IRF Shift Fixed
Initial Tau
2000
2000
Progress
im_group 1
region 1
success % 0
iterations 23
Fit Selected Decay
Fit Dataset

D

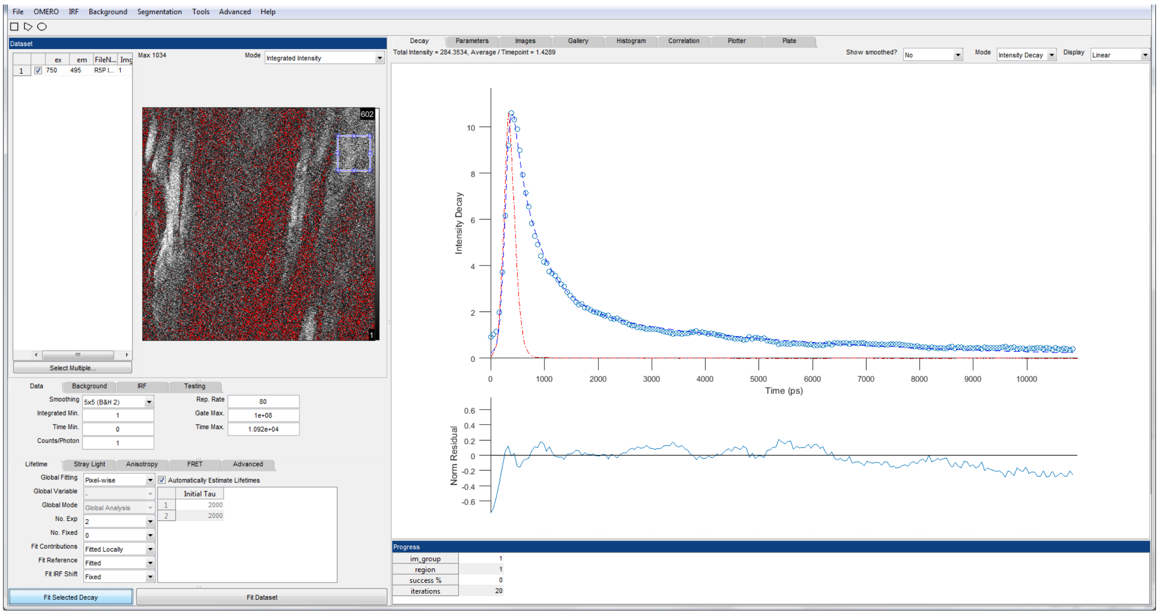

Fig S3. Representative fluorescence lifetime raw curves and fitting results. Data fitting was performed by FLIMfit software tool (*7*) for unglycated (**A, B**) and R5P-glycated collagen (**C,D**) before (**A,C**) and after quenching with triiodide (**B,D**). Open circles – raw data points; dashed line – two-exponential curve fit; red dotted line – instrument response function.

References

1. K. M. Biemel, O. Reihl, M. O. Lederer, Formation Pathways for Lysine-Arginine Cross-links Derived from Hexoses and Pentoses by Maillard Processes. *J. Biol. Chem.* **276**, 23405–23412 (2001).

2. J. R. Lakowicz, *Principles of Fluorescence Spectroscopy* (Springer US, ed. 3, 2006).

3. J. M. Menter, Temperature dependence of collagen fluorescence. *Photochem. Photobiol. Sci.* **5**, 403–410 (2006).

4. K. C. Patra, N. Hay, The pentose phosphate pathway and cancer. *Trends Biochem. Sci.* **39**, 347–354 (2014).

5. J. M. Menter, L. Freeman, O. Edukuye, Thermal and Photochemical Effects on the Fluorescence Properties of Type I Calf Skin Collagen Solutions at Physiological pH, 21–27 (2015).

6. M. E. Westwood, P. J. Thornalley, Molecular characteristics of methylglyoxal-modified bovine and human serum albumins. Comparison with glucose-derived advanced glycation endproduct-modified serum albumins. *J. Protein Chem.* **14**, 359–372 (1995).

7. S. C. Warren *et al.*, Rapid Global Fitting of Large Fluorescence Lifetime Imaging Microscopy Datasets. *PLoS One*. **8** (2013), doi:10.1371/journal.pone.0070687.
